# The Effects of Exercise Training on Functional Aerobic Capacity and Quality of Life in Patients with Systemic Lupus Erythematosus: A Systematic Review of Randomized Controlled Trials

**DOI:** 10.3390/jcm14197031

**Published:** 2025-10-04

**Authors:** Virginia Zouganeli, Stavros Dimopoulos, Alexandros Briasoulis, Achilleas Karkamanis, Panagiotis Panagiotopoulos, Eleftherios Karatzanos, Dimitrios T. Boumpas, Ioannis Vasileiadis, Serafim Nanas, Christos Kourek

**Affiliations:** 1 Clinical Ergospirometry, Exercise & Rehabilitation Laboratory, 1st Critical Care Medicine Department, Evangelismos Hospital, National and Kapodistrian University of Athens, 10676 Athens, Greece; virginia_noa@yahoo.gr (V.Z.); stdimop@gmail.com (S.D.); lkaratzanos@gmail.com (E.K.); ioannisvmed@yahoo.gr (I.V.); sernanas@gmail.com (S.N.); 2 Department of Clinical Therapeutics, National Kapodistrian University of Athens, 11528 Athens, Greece; alexbriasoulis@gmail.com; 3 Department of Cardiology, 417 Army Share Fund Hospital of Athens (NIMTS), 11521 Athens, Greece; achilleaskarkamanis@gmail.com (A.K.); panapopan@gmail.com (P.P.); 4 Rheumatology and Clinical Immunology Unit, “Attikon” University Hospital, National and Kapodistrian University of Athens, 12462 Athens, Greece; boumpasd@uoc.gr

**Keywords:** systemic lupus erythematosus, exercise training, functional capacity, quality of life, outcomes

## Abstract

**Background/Objectives**: Systemic lupus erythematosus (SLE) is associated with impaired functional capacity, persistent fatigue, and poor health-related quality of life despite advances in pharmacological therapy. Exercise training has been proposed as a non-pharmacological intervention, but its efficacy and safety remain incompletely defined. This systematic review aimed to evaluate the effects of exercise training on functional aerobic capacity and quality of life in adults with SLE. **Methods**: A comprehensive search of PubMed, EMBASE, Cochrane Library, and PEDro was conducted to identify randomized controlled trials published up to October 2022, in accordance with the PRISMA guidelines. **Results**: Twelve randomized controlled trials involving 619 participants were included. Exercise interventions were heterogeneous and comprised aerobics, resistance, combined programs, vibration training, home-based protocols, and counseling strategies, with durations ranging from 6 weeks to 12 months. Supervised aerobic and combined interventions consistently improved functional aerobic capacity, while quality of life benefits were reported across several domains, particularly physical health, vitality, and fatigue. Additional positive effects were observed on fatigue, depression, pain, sleep, insulin sensitivity, and self-care ability, without evidence of increased disease activity. **Conclusions**: Structured exercise is safe and can meaningfully enhance functional capacity and quality of life in patients with SLE, supporting its incorporation into multidisciplinary clinical management.

## 1. Introduction

Systemic lupus erythematosus (SLE) is a chronic autoimmune disease characterized by multisystem involvement, fluctuating disease activity, and a relapsing–remitting course [1]. Patients with SLE frequently experience fatigue [2], exercise intolerance [3], musculoskeletal impairment [4], and reduced health-related quality of life [2,4]. In addition, cardiovascular complications and reduced physical fitness contribute to long-term morbidity and mortality [5,6]. Although advances in pharmacological therapy have improved survival, residual symptoms and impaired functional capacity remain major challenges in disease management.

Exercise training is a cornerstone of non-pharmacological management in several chronic inflammatory and cardiovascular conditions [7,8]. Aerobic and resistance training have been shown to improve cardiorespiratory fitness, metabolic health, and psychosocial well-being in patients with rheumatoid arthritis [9], heart failure [10,11], and chronic obstructive pulmonary disease [12]. In SLE, however, concerns regarding disease exacerbation, joint pain, and fatigue have historically limited the widespread adoption of structured exercise interventions. Recent evidence suggests that appropriately prescribed exercise may be both safe and effective [13,14], yet the heterogeneity of interventions and outcome measures complicates interpretation.

Several trials have evaluated different exercise modalities in patients with SLE, including supervised aerobic training [15,16,17,18,19,20,21], resistance programs [15,16,19,21,22], home-based interventions [23,24], vibration exercise [25], and counseling-based approaches supported by digital tools [23,26]. These studies have investigated a broad range of outcomes such as aerobic capacity, fatigue, muscle strength, pain, mental health, self-care, and health-related quality of life. While individual trials have demonstrated promising results, the overall effect of exercise on functional aerobic capacity and quality of life in SLE has not been systematically synthesized.

Given the clinical importance of exercise as a modifiable factor, and the lack of consolidated evidence in SLE, a systematic appraisal of the literature is needed. This review evaluates the effects of exercise training on functional aerobic capacity and quality of life in adult patients with SLE, aiming to provide clinicians and researchers with a clearer understanding of its role, highlight areas of consistent benefit, and identify gaps requiring further investigation.

## 2. Materials and Methods

### 2.1. Search Strategy

A comprehensive literature search was performed in PubMed, Cochrane Library, EMBASE, and PEDro to identify randomized controlled trials (RCTs) investigating the effects of exercise interventions in adults with systemic lupus erythematosus (SLE). The search covered publications from 1982 to October 2022 and used the following terms: (“systemic lupus erythematosus” OR “lupus” OR “SLE”) AND (“exercise” OR “exercise training” OR “rehabilitation” OR “breathing exercise”). Only articles published in English were considered. Duplicate records were removed, and the remaining studies were screened independently by two reviewers based on title and abstract, followed by full-text assessment. Disagreements were resolved by consensus with a third reviewer. This systematic review was conducted and reported in accordance with the Preferred Reporting Items for Systematic Reviews and Meta-Analyses (PRISMA) 2020 guidelines.

### 2.2. Eligibility Criteria

Studies were eligible if they met the following criteria:

1. Design: RCTs with an intervention and a control group.
2. Population: adult patients (≥18 years) with a confirmed diagnosis of SLE under stable treatment.
3. Intervention: structured exercise training programs, including aerobic, resistance, combined, or alternative exercise modalities (vibration, home-based, or counseling).
4. Duration: interventions lasting at least 2 weeks.
5. Outcomes: reporting on functional aerobic capacity [peak oxygen consumption (VO_2_), walking test, ergospirometry, chronotropic reserve, or step count] and/or quality of life assessed by validated questionnaires [36-item Short Form Health Survey questionnaire (SF-36), LupusQoL, Health Assessment Questionnaire (HAQ)].

Exclusion criteria included systematic reviews, meta-analyses, observational or acute exercise studies, and trials including patients with severe comorbidities such as advanced heart failure or chronic obstructive pulmonary disease. Moreover, articles for which full text was not publicly available or accessible through institutional resources were excluded from the review.

### 2.3. Data Extraction

From each study we extracted: (a) author, year, and country; (b) sample size and baseline characteristics [age, sex, disease duration, Systemic Lupus Erythematosus Disease Activity Index (SLEDAI)]; (c) details of the intervention protocol (type, frequency, duration, and intensity); (d) comparator group; (e) outcomes measured; and (f) main findings. Information was tabulated (Table 1 and Table 2) to allow comparison of study characteristics and results.

### 2.4. Risk of Bias Assessment

The methodological quality of included RCTs was assessed using the Cochrane risk-of-bias tool (RoB-2), applied separately for functional aerobic capacity and quality-of-life outcomes. Each study was rated across five domains (randomization, deviations from intended interventions, missing outcome data, outcome measurement, and selective reporting), with overall risk categorized as low, intermediate, or high. Assessments were performed independently by two reviewers, with disagreements resolved by consensus.

### 2.5. Primary and Secondary Outcomes

The primary outcomes of interest were functional aerobic capacity, expressed through peak VO_2_, 6 min walk test, ergospirometry, step counts, or related measures of cardiorespiratory fitness, and/or quality of life, evaluated by validated self-report tools such as the SF-36, LupusQoL, or HAQ.

The secondary outcomes included other patient- or disease-related variables reported across the trials, such as i. fatigue [Fatigue Severity Scale (FSS), Functional Assessment of Chronic Illness Therapy-Fatigue (FACIT-F) scale, SF-36 vitality domain], ii. depression and mood [Beck Depression Inventory (BDI), Patient Health Questionnaire-9 (PHQ-9)], iii. pain [visual analog scale (VAS), McGill Pain Questionnaire (MPQ)], iv. sleep quality [Pittsburgh Sleep Quality Index (PSQI)], v. functional ability and daily activities [HAQ, Disabilities of the Arm, Shoulder and Hand (DASH), hand strength and dexterity tests, Timed Up and Go test (TUG)], vi. metabolic and laboratory outcomes [insulin sensitivity, fasting insulin, Homeostasis Model Assessment—Insulin Resistance (HOMA-IR), Matsuda index, AMP-activated protein kinase (AMPK)/glucose transporter type 4 (GLUT4) expression in muscle biopsy], vii. disease activity indices [SLEDAI, Systemic Lupus International Collaborating Clinics/American College of Rheumatology (SLICC/ACR) Damage Index (SDI)], viii. hospital readmissions and self-care ability (ESCA), and/or ix. lifestyle/behavioral measures (physical activity levels, sedentary time, self-management and habit scores).

Primary and secondary outcomes were assessed at baseline and after the exercise training program.

## 3. Results

### 3.1. Study Selection Results

The database search yielded 1243 records (PubMed = 512, EMBASE = 436, Cochrane = 195, PEDro = 100). After removal of 327 duplicates, 916 records remained for screening. Following title and abstract screening, 874 articles were excluded because they did not meet inclusion criteria (reviews, observational studies, acute exercise protocols, non-SLE populations, or irrelevant outcomes).

**Figure 1:**
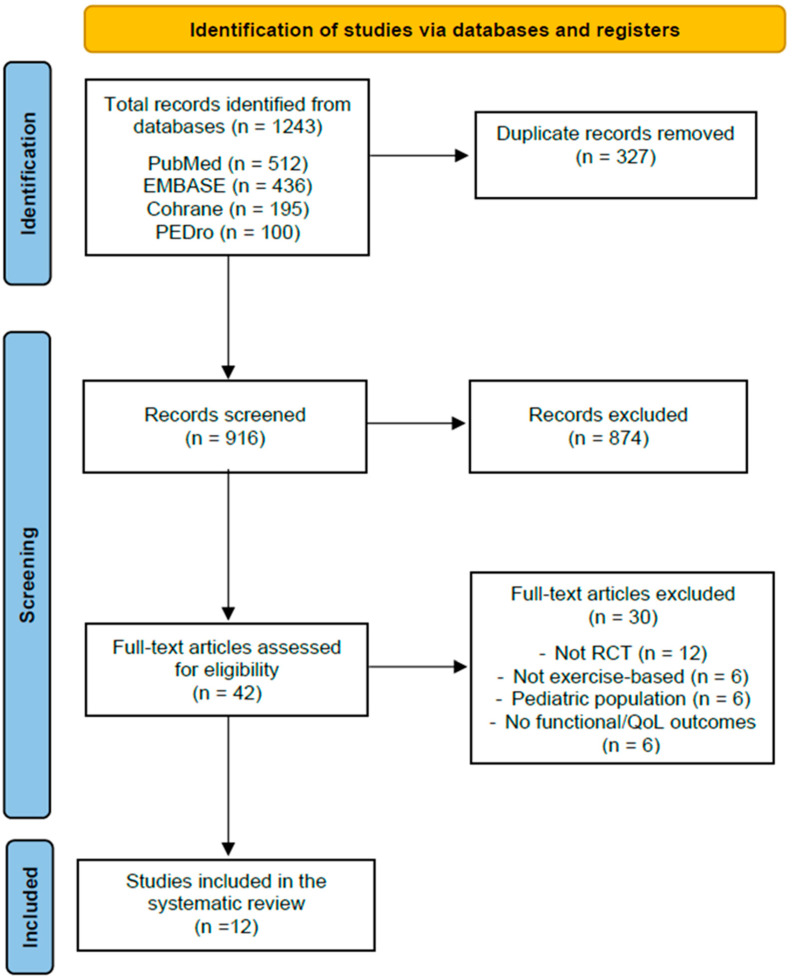
PRISMA flow diagram of the study selection process.

The full text of 42 articles was assessed for eligibility. Of these, 30 studies were excluded for the following reasons: not randomized controlled design (n = 12), intervention not exercise-based (n = 6), pediatric populations (n = 6) and absence of functional aerobic capacity or QoL outcomes (n = 6). Finally, 12 RCTs met all inclusion criteria and were included in the systematic review (Figure 1) [15,16,17,18,19,20,21,22,23,24,25,26].

### 3.2. Study and Patient Characteristics

The 12 RCTs included a total of 619 patients with SLE and 8 healthy individuals, with sample sizes ranging from 19 to 125 participants. Among them, 414 SLE patients performed exercise training while the rest received usual care. The majority of participants were women (≈92–100%). Mean age across studies varied between 31 and 53 years, and disease duration ranged from 3 to 21 years. Baseline SLEDAI scores reflected mostly mild-to-moderate disease activity. Table 1 summarizes the main baseline characteristics of the included patients.

### 3.3. Interventions

Exercise interventions were heterogeneous and included aerobic training, resistance training, combined aerobic and resistance protocols, home-based strengthening/stretching, whole-body vibration, and counseling-based physical activity programs with or without wearable devices. Training duration ranged from 6 weeks to 12 months, with frequencies between 2 and 5 sessions per week. Intervention details, outcomes, and main results are presented in Table 2.

### 3.4. Outcomes

#### 3.4.1. Functional Aerobic Capacity

Nine of the included trials assessed parameters of functional aerobic capacity (Table 2). Supervised aerobic or combined exercise programs were generally associated with significant improvements. In the study by Miossi et al. [21], supervised aerobic and resistance training enhanced peak VO_2_, chronotropic reserve, and heart rate recovery, leading to values comparable to healthy controls. Similarly, Abrahão et al. [15] demonstrated that cardiovascular training improved walking distance in the 12 min test compared with resistance training and usual care. Counseling-based interventions also showed positive effects. Wu et al. [23] reported significant increases in daily step counts, while Li et al. [26] observed trends towards increased moderate-to-vigorous physical activity; however, the latter effect was more evident in rheumatoid arthritis patients than in those with SLE. Carvalho et al. [20] showed that exercise training improved VO_2_ max and exercise tolerance in patients compared to controls. Other interventions produced more modest results. Boström et al. [19] found that VO_2_ max increased over time in both the exercise and control groups, without significant between-group differences, and Avaux et al. [16] reported no improvement in workload capacity despite a reduction in fatigue. Taken together, these findings suggest that structured, supervised exercise interventions are more effective in improving functional aerobic capacity, whereas home-based or counseling programs yield less consistent outcomes.

#### 3.4.2. Quality of Life

Ten trials examined quality of life, primarily through the SF-36 or LupusQoL instruments (Table 2). Improvements were noted in several domains, although results varied between studies. Abrahão et al. [15] found that both cardiovascular and resistance training improved SF-36 scores, with the most pronounced effects in the cardiovascular training group. Bogdanovic et al. [18] demonstrated that both aerobic and isotonic training improved all SF-36 domains and reduced fatigue and depression, while Keramiotou et al. [22] reported gains in physical health and fatigue domains of the LupusQoL following a structured upper-limb program. Wu et al. [23] observed increases in vitality and mental health scores that correlated with daily step counts, and Xie et al. [24] showed that transitional care interventions significantly improved both physical and mental component summaries of the SF-36 and reduced hospital readmissions. Finally, Carvalho et al. [20] showed that patients who performed exercise training improved SF-36 aspects compared to controls. Conversely, some interventions showed limited effects; Boström et al. [19] reported an improvement in mental health at six months that was not sustained over longer follow-up, while Lopes-Souza et al. [25] did not detect changes in SF-36 despite improvements in functional ability. Overall, exercise interventions were associated with modest but consistent improvements in quality of life, particularly in physical health, vitality, and fatigue-related domains.

#### 3.4.3. Secondary Outcomes

Beyond functional capacity and quality of life, several secondary outcomes were reported across the included trials (Table 2). Fatigue was consistently improved in both supervised and home-based interventions, as demonstrated by Avaux et al. [16] and Bogdanovic et al. [18], although findings were not uniform, with Lopes-Souza et al. [25] reporting no benefit in fatigue despite improvements in functional ability. Psychological outcomes also showed favorable effects, with reductions in depression following isotonic and aerobic training, and improvements in mood scores in counseling-based programs. Pain was alleviated in studies incorporating upper limb strengthening and self-management interventions, while Wu et al. [23] reported better sleep quality in parallel with increased physical activity. Importantly, Xie et al. [24] showed that transitional care programs enhanced self-care ability and reduced hospital readmissions, underscoring the potential role of exercise-related interventions in broader health outcomes. At the mechanistic level, Benatti et al. [17] demonstrated that exercise enhanced insulin sensitivity and promoted beneficial skeletal muscle adaptations. Across all studies, disease activity indices remained stable, indicating that exercise training was safe and did not exacerbate SLE activity. Finally, Carvalho et al. [20] demonstrated significant improvements in oxygen pulse, HAQ, fatigue and depression after exercise, compared to controls.

### 3.5. Risk of Bias

The overall methodological quality of the included trials was variable. Randomization and allocation procedures were generally adequate, but lack of blinding of participants and reliance on self-reported QoL outcomes introduced potential bias. Risk of bias was judged as low to intermediate for functional capacity in most exercise training studies, while QoL outcomes were often rated at high risk of bias due to self-reporting without blinding (Table 3).

## 4. Discussion

In this systematic review, evidence derived exclusively from randomized controlled trials investigating the effects of structured exercise interventions in SLE was synthesized. Across a variety of exercise modalities, it was consistently observed that improvements in functional aerobic capacity and quality of life could be achieved without provoking disease exacerbation. These findings provide important reassurance regarding the safety profile of exercise training in a population traditionally considered vulnerable to disease flares and exercise intolerance. By evaluating both functional aerobic capacity and patient-reported quality of life simultaneously, this review adds new insights to the literature, as most previous reports have focused on only one of these dimensions or have included heterogeneous study designs that limited the strength of conclusions. The exclusive inclusion of randomized controlled trials also enhances the reliability of the evidence, thereby filling an important gap in the field.

The clinical relevance of these findings is considerable. Patients with SLE continue to experience impaired functional status, fatigue, and diminished quality of life despite advances in pharmacological therapy [2,3,4]. Cardiovascular morbidity, reduced exercise tolerance, and psychosocial impairment remain major contributors to poor outcomes in this disease. The demonstration that structured exercise programs can ameliorate functional capacity and health-related quality of life suggests that exercise should no longer be viewed as an optional adjunct, but rather as an integral component of comprehensive care. Implementation of supervised aerobic or combined training into standard clinical practice has the potential to enhance daily functioning, reduce long-term disability, and improve overall well-being [27,28]. Furthermore, these findings highlight the role of exercise as a modifiable lifestyle factor that may empower patients to actively participate in their own disease management. Incorporating tailored exercise prescriptions into rheumatology practice, supported by multidisciplinary teams including physiotherapists and exercise specialists, could therefore represent a meaningful step forward in optimizing care for individuals living with SLE.

In relation to prior literature, the present review extends and refines earlier syntheses by restricting inclusion to randomized controlled trials and by jointly evaluating functional aerobic capacity and health-related quality of life as co-primary outcomes. Earlier systematic reviews have provided valuable insights but often combined heterogeneous study designs or focused primarily on fatigue and other patient-reported outcomes, without a parallel appraisal of objective measures of cardiorespiratory fitness [14,29,30]. Consequently, conclusions regarding exercise efficacy in SLE were tentative. By incorporating trials that used rigorous cardiopulmonary testing (VO_2_-based indices, walk tests) alongside validated QoL instruments, and by adding more recent studies that include supervised training, home-based or counseling interventions, and technology-supported activity monitoring, a more coherent and clinically actionable picture has been provided. Importantly, outcome-specific RoB 2 judgments were applied, clarifying that functional capacity evidence is generally more robust than quality-of-life evidence, which is vulnerable to expectancy effects in unblinded settings. Taken together, the available randomized data support the safety and benefit of structured exercise in SLE and delineate where confidence is highest, thereby advancing the field beyond prior narrative summaries and mixed-design meta-analyses.

Exercise intolerance in SLE is multifactorial and reflects the combined effects of chronic inflammation, immune-mediated tissue damage, and treatment-related adverse effects [31]. Persistent systemic inflammation in chronic conditions contributes to endothelial dysfunction, arterial stiffness, and accelerated atherosclerosis, which impair oxygen delivery to working muscles and increase cardiovascular risk [32,33,34,35]. Skeletal muscle is also directly affected, with mitochondrial abnormalities, reduced oxidative capacity, and fiber atrophy leading to early fatigue and diminished exercise tolerance [36,37,38,39]. Corticosteroid therapy, while effective in controlling disease activity, promotes sarcopenia, myopathy, and weight gain, further compounding physical limitations [40,41]. In addition, anemia of chronic disease and autonomic dysfunction may blunt cardiorespiratory responses to exertion, reducing peak oxygen consumption and recovery capacity [42,43]. These physiological deficits are often accompanied by musculoskeletal pain, joint stiffness, and overwhelming fatigue, which discourage participation in regular activity [4,44]. The consequence is a cycle of physical deconditioning and reduced functional reserve, which directly translates into impaired performance of daily tasks and diminished independence [45,46]. Beyond the physical consequences, exercise intolerance exerts a profound psychosocial burden, as limitations in mobility, persistent fatigue, and reduced participation in social and occupational roles contribute to poor health-related quality of life [2,4]. This close interplay between physiological dysfunction and psychosocial impairment underscores why interventions aimed at restoring functional capacity are central not only to physical health but also to the overall well-being of patients with SLE.

Exercise training has the potential to be beneficial for many of the physiological disturbances that underlie exercise intolerance in SLE. Regular aerobic activity promotes endothelial function by enhancing nitric oxide bioavailability [47,48,49], reducing oxidative stress [50,51,52], and improving vascular reactivity [53,54], thereby facilitating oxygen delivery to peripheral tissues. Skeletal muscle adaptations, including increased mitochondrial density, enhanced oxidative enzyme activity, and improved capillary supply, restore metabolic efficiency and delay the onset of fatigue [55,56,57]. Resistance and combined training mitigate corticosteroid-induced sarcopenia and myopathy by preserving lean body mass and increasing muscular strength [58,59,60], which translates into better performance of daily activities. Exercise has also been shown to modulate systemic inflammation by reducing pro-inflammatory cytokines and enhancing regulatory immune pathways, potentially contributing to improved disease control [61,62,63,64]. Beyond these physiological benefits, exercise positively influences psychosocial outcomes: reductions in fatigue, depressive symptoms, and pain have been consistently observed, while improvements in self-efficacy and social participation enhance perceived quality of life [15,16,17,18,19,20,21,22,23,24,25,26]. Collectively, these adaptations suggest that structured exercise represents not only a safe adjunct to pharmacological therapy but also a targeted strategy to interrupt the cycle of deconditioning, functional decline, and poor well-being that characterizes SLE.

Several limitations of this review should be acknowledged. The number of available randomized controlled trials in systemic lupus erythematosus remains limited, and sample sizes across individual studies were generally small, which reduces statistical power and may restrict the generalizability of findings. Considerable heterogeneity was present in terms of exercise modality, duration, and intensity, as well as in the tools used to assess functional aerobic capacity and quality of life, making direct comparisons across trials challenging. This heterogeneity complicates direct comparisons or pooling across studies. Differences in instrument sensitivity, cultural adaptation, or validation of scales may also introduce measurement bias, particularly when applying tools in populations not originally validated for those groups. This is especially relevant in cross-cultural settings or studies enrolling participants from different linguistic/ethnic backgrounds. Risk of bias was frequently rated as intermediate or high for patient-reported outcomes, since blinding of participants was not feasible and quality-of-life measures are inherently subjective. Finally, only studies published in English and with full-text availability were included, raising the possibility of language and accessibility bias.

Many of the included trials provided only limited details on baseline SLE manifestations (organ involvement, duration of disease, flare history, serologic features). This hampers our ability to stratify the observed effects of exercise according to disease phenotype. For example, patients with lupus nephritis, central nervous system involvement, or more aggressive serologies may respond differently to physical activity than those with primarily musculoskeletal or mucocutaneous disease; yet the source trials largely lacked the granularity needed for subgroup analyses. Because of this, potential effect modification, or differential benefit, according to disease subtype remains unexplored in our review.

Another important limitation is the paucity of representation of diverse ethnic and racial populations in the included studies. SLE is known to vary in prevalence, severity, and clinical expression across ethnic groups [65]. For instance, Black, Hispanic, and Asian patients often experience more severe disease courses, earlier organ damage, higher rates of nephritis and increased mortality compared to White patients [66]. Without adequate inclusion of these populations, the external validity of our findings is limited. In particular, it is unknown if the magnitude of exercise-related improvements in quality of life, fatigue, or functional capacity would differ across ethnic groups.

Moreover, racial and ethnic disparities in SLE outcomes are not solely biologic, as they are also shaped by social determinants of health (SDOH), including access to care, socioeconomic status, education, health literacy, insurance status, and exposure to systemic biases [67]. Because most trials did not report or adjust for these variables, there is a risk of confounding: for example, participants from more privileged backgrounds may have had better resources (nutrition, baseline fitness, adherence support) that amplify the benefits of exercise interventions.

Many studies examined the effects of physical exercise over relatively short follow-up periods (12–24 weeks). The durability of beneficial effects beyond the intervention period is uncertain. Potential long-term adverse effects or sustainability of functional capacity and/or quality of life remain unexplored in the existing literature.

## 5. Conclusions

This systematic review of randomized controlled trials demonstrates that structured exercise interventions are safe and beneficial in adults with SLE. Across diverse modalities, including aerobics, resistance, combined, vibration, and counseling-based programs, exercise training improved functional aerobic capacity and produced consistent gains in health-related quality of life without increasing disease activity. Additional benefits were observed in fatigue, mood, pain, self-care ability, and metabolic health. These findings support the integration of tailored exercise programs into routine clinical management as an effective non-pharmacological strategy to address functional limitations and enhance quality of life in this patient population.

## Figures and Tables

**Table 1:** Main baseline characteristics of patients with systemic lupus erythematosus included in the systematic review.

Study	Year	Sample Size (n)	Females [n (%)]	Age (Years)	Disease Duration (Years)	BMI (kg/m^2^)	SLEDAI
Abrahao et al. [15]	2016	• Exercise group 1: 21 SLE patients	61 (96.8) in the total sample	43.8 ± 14.6	4.9 ± 4.3	27.5 ± 10.4	2.3 ± 1.7
		• Exercise group 2: 21 SLE patients		39.1 ± 14.4	3.5 ± 3.3	27.8 ± 11.6	1.4 ± 0.6
		• Control group: 21 SLE patients		46.1 ± 14.1	3.08 ± 1.7	30.9 ± 10.1	1.8 ± 0.6
Avaux et al. [16]	2016	• Exercise group 1: 15 SLE patients	15 (100)	43 ± 7	16 ± 10	NA	3.60 ± 3.87
		• Exercise group 2: 18 SLE patients	16 (88.9)	37 ± 7	12 ± 7	NA	2.33 ± 3.78
		• Control group: 9 SLE patients	9 (100)	46 ± 11	16 ± 10	NA	1.78 ± 2.72
Benatti et al. [17]	2018	• Exercise group: 9 SLE patients	9 (100)	34.8 ± 4.1	9.8 ± 4.1	26.3 ± 3.4	0.22 ± 0.67
		• Control group: 10 SLE patients	10 (100)	32.4 ± 6.5	8.5 ± 5.9	26.2 ± 3.8	0.40 ± 1.26
Bogdanovic et al. [18]	2015	• Exercise group 1: 30 SLE patients	30 (100)	38.8 ± 12.6	5.5 ± 4.1	NA	≤5 in all patients
		• Exercise group 2: 30 SLE patients	30 (100)	47.9 ± 11.5	7.5 ± 6.9	NA	
Bostrom et al. [19]	2016	• Exercise group: 18 SLE patients	18 (100)	52 ± 10	15 ± 9	26.5 ± 5.8	1 (0–8)
		• Control group: 17 SLE patients	17 (100)	53 ± 9	21 ± 14	25.8 ± 3.9	2 (0–3)
Carvalho et al. [20]	2005	• Exercise group: 41 SLE patients	41 (100)	36.22 ± 10.79	5.84 ± 4.84	26.64 ± 4.86	1.15 ± 2.01
		• Control group: 19 SLE patients	19 (100)	35.21 ± 9.13	6.56 ± 3.57	25.81 ± 4.37	1.75 ± 2.45
Keramiotou et al. [22]	2020	• Exercise group: 32 SLE patients	31 (96.9)	43.34 ± 8.90	6 ± 10	NA	4.25 ± 3.24
		• Control group: 30 SLE patients	27 (90)	48.77 ± 12.38	11 ± 15	NA	4.20 ± 3.58
Li et al. [26]	2020	• Exercise immediate group: 16 SLE patients	13 (81.3)	49.9 ± 12.2	NA	28.1 ± 5.5	NA
		• Exercise delayed group: 16 SLE patients	14 (87.5)	47.1 ± 13.8	NA	27.0 ± 10.3	NA
Lopes-Souza et al. [25]	2021	• Exercise group 1: 11 SLE patients	11 (100)	48.5 ± 4.7	13.5 ± 5.2	26.9 ± 5.3	NA
		• Exercise group 2: 10 SLE patients	10 (100)	47.0 ± 7.9	14.8 ± 7.1	24.8 ± 3.3	NA
Miossi et al. [21]	2012	• Training group:14 SLE patients	14 (100)	31.4 ± 5.9	6.1	25.3 ± 4.7	0.9 ± 1.5
		• Non-trained group: 10 SLE patients	10 (100)	31.0 ± 4.8	6.4	23.6 ± 1.9	1.0 ± 1.3
		• Control group: 8 healthy individuals	8 (100)	30.9 ± 8.3	NA	23.9 ± 3.2	NA
Wu et al. [23]	2019	• Exercise group: 38 SLE patients	38 (100)	43.76 ± 9.92	11.97 ± 7.41	22.68 ± 3.98	2.79 ± 2.04
		• Control group: 38 SLE patients	38 (100)	43.45 ± 12.70	12.32 ± 8.42	22.12 ± 3.20	3.39 ± 2.65
Xie et al. [24]	2018	• Exercise group: 64 SLE patients	57 (89.1)	35.9 ± 12.3	≤3 years: 33 (51.6%) >3 years: 31 (48.4%)	NA	10.9 ± 4.9
		• Control group: 61 SLE patients	54 (88.5)	38.4 ± 15.8	≤3 years: 30 (49.2%) >3 years: 31 (50.8%)	NA	9.7 ± 3.8

SLE, systemic lupus erythematosus; BMI, body mass index; SLEDAI, Systemic Lupus Erythematosus Disease Activity Index; NA, not available. SLEDAI values are presented as mean ± SD, except for Boström et al., where values are reported as median (range) according to the original publication.

**Table 2:** PICOS table of the studies included in the systematic review.

Study	Intervention by Study Group	Duration	Outcomes	Main Results
Abrahao et al. [15]	Exercise group 1 (CT, cardiovascular training): walking/bicycle, 65–75% HRR Exercise group 2 (RT, resistance training): weights/bands, 65–75% 1RM, 3 sets of 15 repetitions, 1 min rest between sets Control group: usual care	50 min, 3×/week, 12 weeks	Primary outcome: SF-36 Secondary outcomes: severity of depression (BDI), disease activity (SLEDAI), aerobic capacity (12 min walk test)	- Both CT and RT improved SF-36 vs. baseline; CT superior to RT/control. - Aerobic capacity ↑ in CT vs. RT/control (*p* = 0.001). - No changes in SLEDAI or BDI within exercise groups or compared to the controls.
Avaux et al. [16]	Exercise group 1 (STG, supervised group) and exercise group 2 (HTG, home-based group): mixed endurance/strength training (endurance exercises, walking or bicycle, strengthening exercises with elastoband or weights at 60–80% HRmax) Control group: usual level of physical activity	3 h/week,12 weeks	Primary outcome: change in Krupp’s fatigue severity scale (FSS) Secondary outcomes: aerobic capacity (PWC75%/kg), perceived exertion (Borg scale)	- Both STG (*p* = 0.007) and HTG (*p* = 0.003), but not the CG, statistically improved their FSS at month 3. - The PWC75%/kg and the Borg’s scale did not improve in none of the groups. - Compliance low (~50%) but unrelated to benefit. - Improvement in FSS was sustained after 9 months.
Benatti et al. [17]	Exercise group: supervised aerobic training (5 min warm-up, 30–50 min of treadmill walking, 5 min cooling-down period, moderate intensity) Control group: usual level of physical activity	2×/week, 12 weeks	Primary outcome: Insulin sensitivity (meal test, HOMA-IR, Matsuda index) Secondary outcomes: muscle biopsies (AMPK, GLUT4), aerobic capacity (peak VO_2_, time to exhaustion)	- Exercise improved insulin sensitivity (↓fasting insulin, ↓HOMA-IR, ↑Matsuda index) and increased exercise tolerance (time to exhaustion, ventilatory thresholds) compared to controls. - No change in peak VO_2_ or body composition between groups.
Bogdanovic et al. [18]	Exercise group 1: aerobic training on a bicycle ergometer for 15 min Exercise group 2: isotonic training exercises (increase range of motion and muscle strength), for 30 min, combined with a focus on concentration, balance, breathing and relaxation	30 min, 3×/week, 6 weeks	Fatigue (FSS), depression (BDI), QoL (SF-36)	- Both exercise types significantly reduced fatigue and depression and improved all SF-36 domains. - No significant differences between aerobic vs. isotonic training.
Bostrom et al. [19]	Exercise group: 3 months supervised high-intensity aerobic + strength exercise with coaching (for 60 min), followed by 9 months self-managed activity at low-to-moderate intensity with reduced support Control group: usual care	3×/week, 12 months	Primary outcomes: Aerobic capacity (VO_2_ max, ergometer), physical activity (self-report), QoL (SF-36) Secondary outcomes: Disease activity (SLEDAI), organ damage (SLICC) and pharmacological treatment	- VO_2_ max and submax VO_2_ increased over time in both groups (training effect, no group × time interaction → no differences in physical activity and aerobic capacity between the groups). - Mental health improved at 6 months in exercise group only; no other QoL changes. - There were no baseline differences between groups in SLEDAI, SLICC and pharmacological treatment.
Carvalho et al. [20]	Exercise group: supervised treadmill walking at anaerobic threshold HR for 60 min (10 min warmup/stretching, 40 min walking, and 10 min cooling down) Control group: usual care	3×/week, 12 weeks	Functional capacity (VO_2_ max, anaerobic threshold, exercise tolerance), QoL (SF-36), fatigue, depression, pain, HAQ	- Training improved VO_2_ max, exercise tolerance, oxygen pulse, HAQ, fatigue, depression, and SF-36 (physical, vitality, functional capacity) within exercise group and compared to controls.
Keramiotou et al. [22]	Exercise group: upper limb strengthening/stretching home program by hand therapist (9 strengthening and stretching exercises for the upper extremities with a stick, 10 for the fingers and 11 against resistance with therapeutic putty), plus routine care Control group: routine care + advice	30 min/daily, 12 weeks, follow-up until 24 weeks	Primary outcome: Hand function (DASH score) Secondary outcomes: Hand function (HAQ score, grip/pinch strength, dexterity), QoL (LupusQoL—physical health, fatigue), pain (VAS)	- Exercise group showed significant improvements in daily activity performance, hand strength, dexterity, pain, and LupusQoL domains (physical health, fatigue) at 6, 12 and 24 weeks vs. control. - No interaction was observed between exercise and disease activity or medication.
Li et al. [26]	Exercise immediate group: first 8-week physiotherapist-led counseling + Fitbit + web app feedback + 4 follow-up calls Exercise delayed group: same intervention in weeks 10–17	8 weeks for each group; assessment at baseline, week 9, 18 and 27	Primary outcome: MVPA time by accelerometer Secondary outcomes: Physical activity (steps, sedentary time), pain (MPQ-SF), fatigue (FSS), mood (PHQ-9), self-management, habits	- Both immediate and Delay showed: MVPA↑, daily steps ↑, PHQ-9 ↓, MPQ-SF↓ Partners in Health Scale ↑. - No significant between-group effect on MVPA (trend ↑9.4 min/day). - Post hoc analysis revealed a significant effect in MVPA and pain in participants with rheumatoid arthritis, but not those with SLE.
Lopes-Souza et al. [25]	Exercise group 1: warm-up with no defined load for 2min at the cycle ergometer, whole-body vibration exercise (WBVE) with 130^ο^ knee flexion without hand support, 10 repetitions vibration exposure—30 sec of passive rest Exercise group 2: isometry (same stance without vibration)	2×/week, 12 weeks	Fatigue (FACIT-F), functional ability (HAQ, TUG), QoL (SF-36)	- WBVE group significantly improved HAQ vs. control at 6 and 12 weeks (*p* = 0.03). - No significant differences in fatigue between groups. - No significant changes in TUG or SF-36 domains within and between groups.
Miossi et al. [21]	Training group: supervised combined aerobic (5 min warm up, 30 min treadmill with maximal-graded exercise protocol, 2 min recovery at the initial workload) + resistance training (7 exercises, 4 × 8–12 RM) + 5 min of stretching exercises Non-trained group: usual daily activities Control group: supervised exercise training program	2×/week, 12 weeks	Primary outcomes: Chronotropic reserve, HR recovery [difference between HR at peak exercise and at both the first (HRR1) and second (HRR2) minutes after the exercise test] Secondary outcomes: HRR1, HRR2, Functional aerobic capacity (peak VO_2_), Disease activity (SLEDAI)	-Both SLE groups were comparable at baseline and had lower peak HR, peak VO2, and CR vs. the control group at baseline. - Exercise significantly improved chronotropic reserve, HR recovery, and HR response in both SLE groups vs. the controls. - Neither the non-trained nor the control group presented any change in the CR or in HRR1 and HRR2 (*p* > 0.05). - No significant changes within the control group. - Trained SLE patients achieved values comparable to healthy controls. - SLEDAI remained stable.
Wu et al. [23]	Exercise group: physical activity counseling (5A’s model) with 3 face-to-face and 3 follow-up phone sessions + pedometer monitoring exercise Control group: normal lifestyle and clinical visits as usual	12 weeks	Primary outcome: physical activity (daily steps- pedometer) Secondary outcomes: fatigue (FSS), sleep quality (PSQI), QoL (SF-36)	- Exercise group significantly ↑daily steps (+1309 vs. +286, respectively, *p* < 0.001), improved sleep quality (B = −1.08, *p* < 0.01 and B = −1.24, *p* < 0.01, respectively) and SF-36 vitality (B = 7.20, *p* = 0.01 and B = 9.15, *p* < 0.01, respectively) at 8 and 12 weeks, and mental health at 8 weeks (B = 4.34, *p* < 0.05) compared to the controls. - No effect on fatigue or other SF-36 domains within groups or between groups. - Vitality and mental health score changes showed significant positive correlations with daily step changes (r = 0.49, *p* < 0.01 and r = 0.50, *p* < 0.01, respectively).
Xie et al. [24]	Exercise group: transitional care program (Omaha System framework: 4 face-to-face sessions + 4 phone follow-ups) Control group: usual care	3 months	After 30, 60 and 90 days: Self-care (ESCA), QoL (SF-36 PCS/MCS), readmission rates, disease activity (SLEDAI-2K)	- Transitional care significantly improved self-care (↑ ESCA) and SF-36 PCS/MCS vs. control (*p* < 0.001). - It also reduced 30-, 60-, and 90-day readmissions compared to the controls (*p* = 0.005). - No significant change in SLEDAI-2K within or between groups.

SLE, systemic lupus erythematosus; HRR, heart rate reserve; 1RM; one repetition maximum; SF-36, short form health survey-36; BDI, Beck depression inventory, SLEDAI, systemic lupus erythematosus disease activity index; HRmax, maximum heart rate; FSS, fatigue severity scale; PWC75%/kg, physical work capacity at 75% of predicted HR normalized by weight; AMPK, AMP-activated protein kinase; GLUT4, glucose transporter type 4; VO_2_, oxygen uptake; QoL, quality of life; SLICC, systemic lupus international collaborating clinics damage index; HAQ, health assessment questionnaire; DASH, disabilities of the arm, shoulder, and hand questionnaire; LupusQoL, lupus quality of life questionnaire; VAS, visual analog scale; MVPA, moderate-to-vigorous physical activity; MPQ-SF, McGill pain questionnaire—short form; PHQ-9, patient health questionnaire-9; TUG, timed up and go test; HRR1, HRR2, heart rate recovery at 1 and 2 min after exercise; CR, chronotropic reserve; PSQI, Pittsburgh sleep quality index; 5A’s model, ask, advise, assess, assist, arrange; ESCA, exercise of self-care agency scale; PCS/MCS, physical and mental component summary scores of SF-36; ↑, increase; ↓, decrease.

**Table 3:** Cochrane risk-of-bias tool for assessment of quality of life and functional capacity among randomized trials version 2 (RoB-2).

Study	Risk of Bias Domains					
	Randomization Process	Deviations from Intended Interventions	Missing Outcome Data	Measurement of the Outcome	Selection of the Reported Result	Overall Risk of Bias
***Abrahao* et al.** [15]						
Quality of life	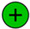	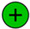	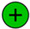	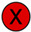	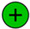	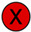
Functional capacity	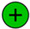	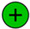	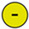	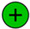	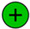	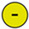
***Avaux* et al.** [16]						
Quality of life	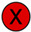	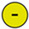	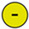	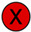	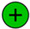	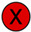
Functional capacity	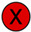	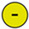	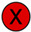	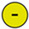	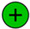	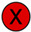
***Benatti* et al.** [17]						
Quality of life	Not assessed					
Functional capacity	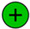	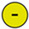	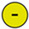	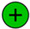	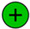	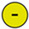
***Bogdanovic* et al.** [18]						
Quality of life	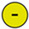	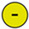	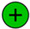	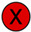	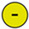	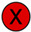
Functional capacity	Not assessed					
***Bostrom* et al.** [19]						
Quality of life	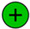	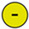	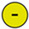	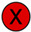	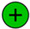	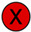
Functional capacity	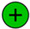	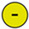	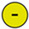	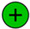	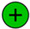	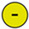
***Carvalho* et al.** [20]						
Quality of life	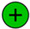	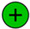	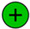	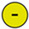	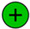	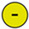
Functional capacity	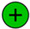	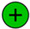	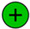	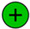	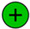	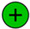
***Keramiotou* et al.** [22]						
Quality of life	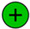	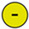	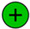	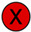	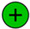	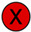
Functional capacity	Not assessed					
***Li* et al.** [26]						
Quality of life	Not assessed					
Functional capacity	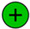	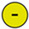	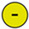	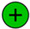	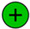	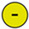
***Lopes-Souza* et al.** [25]						
Quality of life	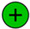	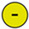	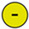	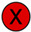	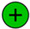	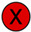
Functional capacity	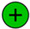	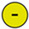	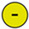	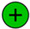	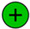	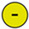
***Miossi* et al.** [21]						
Quality of life	Not assessed					
Functional capacity	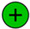	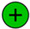	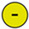	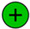	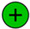	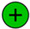
***Wu* et al.** [23]						
Quality of life	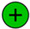	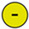	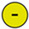	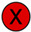	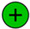	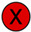
Functional capacity	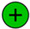	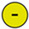	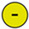	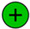	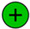	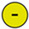
***Xie* et al.** [24]						
Quality of life	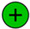	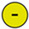	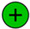	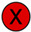	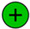	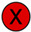
Functional capacity	Not assessed					
Judgement: 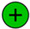 Low 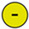 Moderate 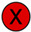 High						

## Abbreviations

The following abbreviations are used in this manuscript:

SLE	Systemic lupus erythematosus
HRR	Heart rate reserve
1RM	One repetition maximum
SF-36	Short form health survey-36
BDI	Beck depression inventory
SLEDAI	Systemic lupus erythematosus disease activity index
HRmax	Maximum heart rate
FSS	Fatigue severity scale
PWC75%/kg	Physical work capacity at 75% of predicted HR normalized by weight
AMPK	AMP-activated protein kinase
GLUT4	Glucose transporter type 4
VO_2_	Oxygen uptake
QoL	Quality of life
SLICC	Systemic lupus international collaborating clinics damage index
HAQ	Health assessment questionnaire
DASH	Disabilities of the arm, shoulder, and hand questionnaire
LupusQoL	Lupus quality of life questionnaire
VAS	Visual analog scale
MVPA	Moderate-to-vigorous physical activity
MPQ-SF	McGill pain questionnaire—short form
PHQ-9	Patient health questionnaire-9
TUG	Timed up and go test
HRR1, HRR2	Heart rate recovery at 1 and 2 min after exercise
CR	Chronotropic reserve
PSQI	Pittsburgh sleep quality index
5A’s model	Ask, advise, assess, assist, arrange
ESCA	Exercise of self-care agency scale
PCS/MCS	Physical and mental component summary scores of SF-36
CT	Cardiovascular training
RT	Resistance training
STG	Supervised group
HTG	Home-based group
BMI	Body mass index
NA	Not available

## References

[1] Ameer M.A., Chaudhry H., Mushtaq J., Khan O.S., Babar M., Hashim T., Zeb S., Tariq M.A., Patlolla S.R., Ali J. (2022). An overview of systemic lupus erythematosus (SLE) pathogenesis, classification, and management. Cureus.

[2] Nikolopoulos D., Cetrez N., Lindblom J., Palazzo L., Enman Y., Parodis I. (2024). Patients with NPSLE experience poorer HRQoL and more fatigue than SLE patients with no neuropsychiatric involvement, irrespective of neuropsychiatric activity. Rheumatology.

[3] Kim Y.J., Ismail P., Petri M., Fava A., Adamo L. (2025). Preload deficiency as a treatable cause of fatigue and exercise intolerance in SLE. Lancet Rheumatol..

[4] Tharwat S., Husain S.M. (2024). Musculoskeletal symptoms in systemic lupus erythematosus patients and their impact on health-related quality of life. BMC Musculoskelet. Disord..

[5] Alghareeb R., Hussain A., Maheshwari M.V., Khalid N., Patel P.D. (2022). Cardiovascular complications in systemic lupus erythematosus. Cureus.

[6] Atzeni F., Rodríguez-Pintó I., Cervera R. (2024). Cardiovascular disease risk in systemic lupus erythematous: Certainties and controversies. Autoimmun. Rev..

[7] Fairag M., Alzahrani S.A., Alshehri N., Alamoudi A.O., Alkheriji Y., Alzahrani O.A., Alomari A.M., Alzahrani Y.A., Alghamdi S.M., Fayraq A. (2024). Exercise as a therapeutic intervention for chronic disease management: A comprehensive review. Cureus.

[8] Beavers K.M., Brinkley T.E., Nicklas B.J. (2010). Effect of exercise training on chronic inflammation. Clin. Chim. Acta.

[9] Athanasiou A., Papazachou O., Rovina N., Nanas S., Dimopoulos S., Kourek C. (2024). The effects of exercise training on functional capacity and quality of life in patients with rheumatoid arthritis: A systematic review. J. Cardiovasc. Dev. Dis..

[10] Kourek C., Alshamari M., Mitsiou G., Psarra K., Delis D., Linardatou V., Pittaras T., Ntalianis A., Papadopoulos C., Panagopoulou N. (2020). The acute and long-term effects of a cardiac rehabilitation program on endothelial progenitor cells in chronic heart failure patients: Comparing two different exercise training protocols. Int. J. Cardiol. Heart Vasc..

[11] Kourek C., Briasoulis A., Karatzanos E., Zouganeli V., Psarra K., Pratikaki M., Alevra-Prokopiou A., Skoularigis J., Xanthopoulos A., Nanas S. (2023). The effects of a cardiac rehabilitation program on endothelial progenitor cells and inflammatory profile in patients with chronic heart failure of different severity. J. Clin. Med..

[12] Fastenau A., van Schayck O.C., Winkens B., Aretz K., Gosselink R., Muris J.W. (2020). Effectiveness of an exercise training programme for COPD in primary care: A randomized controlled trial. Respir. Med..

[13] Blaess J., Geneton S., Goepfert T., Appenzeller S., Bordier G., Davergne T., Fuentes Y., Haglo H., Hambly K., Kinnett-Hopkins D. (2024). Recommendations for physical activity and exercise in persons living with systemic lupus erythematosus (SLE): Consensus by an international task force. RMD Open.

[14] Blaess J., Goepfert T., Geneton S., Irenee E., Gerard H., Taesch F., Sordet C., Arnaud L. (2023). Benefits and risks of physical activity in patients with systemic lupus erythematosus: A systematic review of the literature. Semin. Arthritis Rheum..

[15] Abrahão M.I., Gomiero A.B., Peccin M.S., Grande A.J., Trevisani V.F. (2016). Cardiovascular training vs. resistance training for improving quality of life and physical function in patients with systemic lupus erythematosus: A randomized controlled trial. Scand. J. Rheumatol..

[16] Avaux M., Hoellinger P., Nieuwland-Husson S., Fraselle V., Depresseux G., Houssiau F.A. (2016). Effects of two different exercise programs on chronic fatigue in lupus patients. Acta Clin. Belg..

[17] Benatti F.B., Miyake C.N.H., Dantas W.S., Zambelli V.O., Shinjo S.K., Pereira R.M.R., Silva M.E.R., Sá-Pinto A.L., Borba E., Bonfá E. (2018). Exercise increases insulin sensitivity and skeletal muscle AMPK expression in systemic lupus erythematosus: A randomized controlled trial. Front. Immunol..

[18] Bogdanovic G., Stojanovich L., Djokovic A., Stanisavljevic N. (2015). Physical activity program is helpful for improving quality of life in patients with systemic lupus erythematosus. Tohoku J. Exp. Med..

[19] Boström C., Elfving B., Dupré B., Opava C.H., Lundberg I.E., Jansson E. (2016). Effects of a one-year physical activity programme for women with systemic lupus erythematosus: A randomized controlled study. Lupus.

[20] Carvalho M.R., Sato E.I., Tebexreni A.S., Heidecher R.T., Schenkman S., Neto T.L. (2005). Effects of a supervised cardiovascular training program on exercise tolerance, aerobic capacity, and quality of life in patients with systemic lupus erythematosus. Arthritis Rheum..

[21] Miossi R., Benatti F.B., Lúcia de Sá Pinto A., Lima F.R., Borba E.F., Prado D.M., Perandini L.A., Gualano B., Bonfá E., Roschel H. (2012). Using exercise training to counterbalance chronotropic incompetence and delayed heart rate recovery in systemic lupus erythematosus: A randomized trial. Arthritis Care Res..

[22] Keramiotou K., Anagnostou C., Kataxaki E., Galanos A., Sfikakis P.P., Tektonidou M.G. (2020). The impact of upper limb exercise on function, daily activities and quality of life in systemic lupus erythematosus: A pilot randomised controlled trial. RMD Open.

[23] Wu M.L., Tsai J.C., Yu K.H., Chen J.J. (2019). Effects of physical activity counselling in women with systemic lupus erythematosus: A randomized controlled trial. Int. J. Nurs. Pract..

[24] Xie X., Song Y., Yang H., Nie A., Chen H., Li J.P. (2018). Effects of transitional care on self-care, readmission rates, and quality of life in adult patients with systemic lupus erythematosus: A randomized controlled trial. Arthritis Res. Ther..

[25] Lopes-Souza P., Dionello C.F., Bernardes-Oliveira C.L., Moreira-Marconi E., Marchon R.M., Teixeira-Silva Y., Paineiras-Domingos L.L., da Cunha Sá-Caputo D., Xavier V.L., Bergmann A. (2021). Effects of 12-week whole-body vibration exercise on fatigue, functional ability and quality of life in women with systemic lupus erythematosus: A randomized controlled trial. J. Bodyw. Mov. Ther..

[26] Li L.C., Feehan L.M., Xie H., Lu N., Shaw C., Gromala D., Aviña-Zubieta J.A., Koehn C., Hoens A.M., English K. (2020). Efficacy of a physical activity counseling program with use of a wearable tracker in people with inflammatory arthritis: A randomized controlled trial. Arthritis Care Res..

[27] Collins K.A., Fos L.B., Ross L.M., Slentz C.A., Davis P.G., Willis L.H., Piner L.W., Bateman L.A., Houmard J.A., Kraus W.E. (2021). Aerobic, resistance, and combination training on health-related quality of life: The STRRIDE-AT/RT randomized trial. Front. Sports Act. Living.

[28] Poli L., Greco G., Cataldi S., Ciccone M.M., De Giosa A., Fischetti F. (2024). Multicomponent versus aerobic exercise intervention: Effects on hemodynamic, physical fitness and quality of life in adult and elderly cardiovascular disease patients: A randomized controlled study. Heliyon.

[29] Vandenbulcke L., Erard M., Van Assche D., De Langhe E. (2023). The effect of physical exercise on fatigue in systemic lupus erythematosus: A systematic review. Acta Clin. Belg..

[30] Lu M.C., Koo M. (2021). Effects of exercise intervention on health-related quality of life in patients with systemic lupus erythematosus: A systematic review and meta-analysis of controlled trials. Healthcare.

[31] Kaul A., Gordon C., Crow M.K., Touma Z., Urowitz M.B., van Vollenhoven R., Ruiz-Irastorza G., Hughes G. (2016). Systemic lupus erythematosus. Nat. Rev. Dis. Primers.

[32] Gusev E., Sarapultsev A. (2023). Atherosclerosis and inflammation: Insights from the theory of general pathological processes. Int. J. Mol. Sci..

[33] Triantafyllias K., Thiele L.E., Cavagna L., Baraliakos X., Bertsias G., Schwarting A. (2023). Arterial stiffness as a surrogate marker of cardiovascular disease and atherosclerosis in patients with arthritides and connective tissue diseases: A literature review. Diagnostics.

[34] Baaten C.C.F.M.J., Vondenhoff S., Noels H. (2023). Endothelial cell dysfunction and increased cardiovascular risk in patients with chronic kidney disease. Circ. Res..

[35] Cacciatore S., Andaloro S., Bernardi M., Oterino Manzanas A., Spadafora L., Figliozzi S., Asher E., Rana J.S., Ecarnot F., Gragnano F. (2025). Chronic inflammatory diseases and cardiovascular risk: Current insights and future strategies for optimal management. Int. J. Mol. Sci..

[36] Lewsey S.C., Weiss K., Schär M., Zhang Y., Bottomley P.A., Samuel T.J., Xue Q.L., Steinberg A., Walston J.D., Gerstenblith G. (2020). Exercise intolerance and rapid skeletal muscle energetic decline in human age-associated frailty. JCI Insight.

[37] Lv J., Li Y., Shi S., Xu X., Wu H., Zhang B., Song Q. (2022). Skeletal muscle mitochondrial remodeling in heart failure: An update on mechanisms and therapeutic opportunities. Biomed. Pharmacother..

[38] Chen X., Ji Y., Liu R., Zhu X., Wang K., Yang X., Liu B., Gao Z., Huang Y., Shen Y. (2023). Mitochondrial dysfunction: Roles in skeletal muscle atrophy. J. Transl. Med..

[39] Keller-Ross M.L., Larson M., Johnson B.D. (2019). Skeletal muscle fatigability in heart failure. Front. Physiol..

[40] Surmachevska N., Tiwari V. (2025). Corticosteroid-Induced Myopathy. StatPearls.

[41] Kuzuya M. (2024). Drug-related sarcopenia as a secondary sarcopenia. Geriatr. Gerontol. Int..

[42] Miliotis P.G., Ntalapera S.D., Lakeas P., Loukas I., Toubekis A.G., Geladas N.D., Koskolou M.D. (2025). Cardiorespiratory and oxygenation responses in iron-deficient anemic women during whole-body exercise under moderate hypoxia. Eur. J. Appl. Physiol..

[43] Listerman J., Geisberg C., Nading M.A., Goring J., Huang R., Butler J. (2007). Blunted hemodynamic response and reduced oxygen delivery with exercise in anemic heart failure patients with systolic dysfunction. Congest. Heart Fail..

[44] Margiotta D.P.E., Basta F., Dolcini G., Batani V., Lo Vullo M., Vernuccio A., Navarini L., Afeltra A. (2018). Physical activity and sedentary behavior in patients with systemic lupus erythematosus. PLoS ONE.

[45] Keramiotou K., Anagnostou C., Konstantonis G., Kataxaki E., Sfikakis P.P., Tektonidou M.G. (2021). Impaired hand function and performance in activities of daily living in systemic lupus erythematosus, even in patients achieving lupus low disease activity state (LLDAS). Rheumatol. Adv. Pract..

[46] Wooten L.C., Hasni S., Mikdashi J.A., Keyser R.E. (2023). Cardiorespiratory insufficiency and performance fatigability in women with systemic lupus erythematosus. Cardiopulm. Phys. Ther. J..

[47] Tao X., Chen Y., Zhen K., Ren S., Lv Y., Yu L. (2023). Effect of continuous aerobic exercise on endothelial function: A systematic review and meta-analysis of randomized controlled trials. Front. Physiol..

[48] Green D.J., Maiorana A., O’Driscoll G., Taylor R. (2004). Effect of exercise training on endothelium-derived nitric oxide function in humans. J. Physiol..

[49] Hambrecht R., Adams V., Erbs S., Linke A., Kränkel N., Shu Y., Baither Y., Gielen S., Thiele H., Gummert J.F. (2003). Regular physical activity improves endothelial function in patients with coronary artery disease by increasing phosphorylation of endothelial nitric oxide synthase. Circulation.

[50] Ye Y., Lin H., Wan M., Qiu P., Xia R., He J., Tao J., Chen L., Zheng G. (2021). The effects of aerobic exercise on oxidative stress in older adults: A systematic review and meta-analysis. Front. Physiol..

[51] Robinson A.T., Fancher I.S., Sudhahar V., Bian J.T., Cook M.D., Mahmoud A.M., Ali M.M., Ushio-Fukai M., Brown M.D., Fukai T. (2017). Short-term regular aerobic exercise reduces oxidative stress produced by acute in the adipose microvasculature. Am. J. Physiol. Heart Circ. Physiol..

[52] Thirupathi A., Wang M., Lin J.K., Fekete G., István B., Baker J.S., Gu Y. (2021). Effect of different exercise modalities on oxidative stress: A systematic review. Biomed. Res. Int..

[53] Craighead D.H., Freeberg K.A., Seals D.R. (2019). The protective role of regular aerobic exercise on vascular function with aging. Curr. Opin. Physiol..

[54] Goeder D., Kröpfl J.M., Angst T., Hanssen H., Hauser C., Infanger D., Maurer D., Oberhoffer-Fritz R., Schmidt-Trucksäss A., Königstein K. (2024). VascuFit: Aerobic exercise improves endothelial function independent of cardiovascular risk: A randomized-controlled trial. Atherosclerosis.

[55] Dent J.R., Stocks B., Campelj D.G., Philp A. (2023). Transient changes to metabolic homeostasis initiate mitochondrial adaptation to endurance exercise. Semin. Cell Dev. Biol..

[56] Smith J.A.B., Murach K.A., Dyar K.A., Zierath J.R. (2023). Exercise metabolism and adaptation in skeletal muscle. Nat. Rev. Mol. Cell Biol..

[57] Lundby C., Jacobs R.A. (2016). Adaptations of skeletal muscle mitochondria to exercise training. Exp. Physiol..

[58] Yasuda T. (2022). Selected methods of resistance training for prevention and treatment of sarcopenia. Cells.

[59] Carcelén-Fraile M.D.C., Lorenzo-Nocino M.F., Afanador-Restrepo D.F., Rodríguez-López C., Aibar-Almazán A., Hita-Contreras F., Achalandabaso-Ochoa A., Castellote-Caballero Y. (2023). Effects of different intervention combined with resistance training on musculoskeletal health in older male adults with sarcopenia: A systematic review. Front. Public Health.

[60] Govindasamy K., Rao C.R., Chandrasekaran B., Parpa K., Granacher U. (2025). Effects of resistance training on sarcopenia risk among healthy older adults: A scoping review of physiological mechanisms. Life.

[61] Docherty S., Harley R., McAuley J.J., Crowe L.A.N., Pedret C., Kirwan P.D., Siebert S., Millar N.L. (2022). The effect of exercise on cytokines: Implications for musculoskeletal health: A narrative review. BMC Sports Sci. Med. Rehabil..

[62] Scheffer D.D.L., Latini A. (2020). Exercise-induced immune system response: Anti-inflammatory status on peripheral and central organs. Biochim. Biophys. Acta Mol. Basis Dis..

[63] Magni O., Arnaoutis G., Panagiotakos D. (2025). The impact of exercise on chronic systemic inflammation: A systematic review and meta–meta-analysis. Sport Sci. Health.

[64] Taherkhani S., Suzuki K., Castell L. (2020). A short overview of changes in inflammatory cytokines and oxidative stress in response to physical activity and antioxidant supplementation. Antioxidants.

[65] Lewis M.J., Jawad A.S. (2017). The effect of ethnicity and genetic ancestry on the epidemiology, clinical features and outcome of systemic lupus erythematosus. Rheumatology.

[66] Parodis I., Lanata C., Nikolopoulos D., Blazer A., Yazdany J. (2023). Reframing health disparities in SLE: A critical reassessment of racial and ethnic differences in lupus disease outcomes. Best Pract. Res. Clin. Rheumatol..

[67] Hasan B., Fike A., Hasni S. (2022). Health disparities in systemic lupus erythematosus-a narrative review. Clin. Rheumatol..

